# Supplementary material for: VESPA: an optimized protocol for accurate metabarcoding-based characterization of vertebrate eukaryotic endosymbiont and parasite assemblages
Source: Nat Commun. 2024 Jan 9;15:402. doi: 10.1038/s41467-023-44521-3 (PMC10776621; doi:10.1038/s41467-023-44521-3)
Supplement: Supplementary file 1 — Supplementary Information [file 41467_2023_44521_MOESM1_ESM.pdf]

Supplementary Information- Tables

Supplementary Table 1. 18S primers used in this study (locus specific sequences).

Primer Name	Reference	F/R	Sequence (5′ – 3′)	Region
515f	Bates 2012	F	GTGCCAGCMGCCGCGGTAA	V4
1119r	Bates 2012	R	GGTGCCCTTCCGTCA	V4
18S-EUK581-F	Bower 2004	F	GTGCCAGCAGCCGCG	V4
18S-EUK1134-R	Bower 2004	R	TTTAAGTTTCAGCCTTGCG	V4
TAReuk454FWD1	Stoeck 2010	F	CCAGCASCYGCGGTAATTCC	V4
V4r	Bradley 2016	R	ACTTTCGTTCTTGAT	V4
3NDf	Cavalier-Smith 2009	F	GGCAAGTCTGGTGCCAG	V4
V4_euk_R2	Brate 2010	R	ACGGTATCTRATCRTCTTCG	V4
V4_euk_R1	Brate 2010	R	GACTACGACGGTATCTRATCRTCTTCG	V4
1132mod	Giesen 2018	R	TCCGTCAATTYCTTTAAGT	V4
E572F	Comeau 2011	F	CYGCGGTAATTCCAGCTC	V4
E1009R	Comeau 2011	R	AYGGTATCTRATCRTCTTYG	V4
18SV4_F	DeMone 2020	F	GCCGCGGTAATTCCAGCTC	V4
18SV4_R	DeMone 2020	R	ATYYTTGGCAAATGCTTTCGC	V4
Giardia 18SV4_R	DeMone 2020	R	ATACGGTGGTGTCTGATCGC	V4
F-566	Hadziavdic 2014	F	CAGCAGCCGCGGTAATTCC	V4
R-1200	Hadziavdic 2014	R	CCCGTGTTGAGTCAAATTAAGC	V4
F-574	Hadziavdic 2014	F	GCGGTAATTCCAGCTCCAA	V4
R-952	Hadziavdic 2014	R	TTGGCAAATGCTTTCGC	V4
574	Hugerth 2014	F	CGGTAAYTCCAGCTCYV	V4
1132	Hugerth 2014	R	CCGTCAATTHCTTYAART	V4
616	Hugerth 2014	F	TTAAARVGYTCGTAGTYG	V4
563	Hugerth 2014	F	GCCAGCAVCYGCGGTAAY	V4
G3F1	Krogsgaard 2018	F	GCCAGCAGCCGCGGTAATTC	V4
G3R1	Krogsgaard 2018	R	ACATTCTTGGCAAATGCTTTCGCAG	V4
G4F3	Krogsgaard 2018	F	AGCCGCGGTAATTCCAGCTC	V4
G4R3	Krogsgaard 2018	R	GGTGGTGCCCTTCCGTCAAT	V4
G6F1	Krogsgaard 2018	F	TGGAGGGCAAGTCTGGTGCC	V4
G6R1	Krogsgaard 2018	R	TACGGTATCTGATCGTCTTCGATCCC	V4
18S#1	Machida 2012	F	CTGGTGCCAGCAGCCGCGGYAA	V4

18S#2RC	Machida 2012	R	TCCGTCAATTYCTTTAAGTT	V4
MMSF	Sikder 2020	F	GGTGCCAGCAGCCGCGGTA	V4
MMSR	Sikder 2020	R	CTTTAAGTTTCAGCTTTGC	V4
Nem18SlongF	Wood 2013	F	CAGGGCAAGTCTGGTGCCAGCAGC	V4
Nem18SlongR	Wood 2013	R	GACTTTCGTTCTTGATTAATGAA	V4
Uni18S	Zhan 2013	F	AGGGCAAKYCTGGTGCCAGC	V4
Uni18SR	Zhan 2013	R	GRCGGTATCTRATCGYCTT	V4
9F	This study	F	CTGGTGCCAGCAGCCGCGG	V4
13F	This study	F	TGGTGCCAGCAGCCGCGG	V4
29F	This study	F	AGCAGCCGCGGTAATTCC	V4
2-2bF	This study	F	TGGTGCCAGCASCCGCG	V4
21b8R	This study	R	TCAATTYCTTIAASTTTC	V4
EukA_F	Medlin 1988	F	AACCTGGTTGATCCTGCCAGT	5' terminus
EukB_R	Medlin 1988	R	TGATCCTTCTGCAGGTTCACCTAC	3' terminus
1520_R	Lopez-Garcia 2003	R	CYGCAGGTTCACCTAC	3' terminus
V3Mod_F	This study (modified from Flaherty 2018)	F	CCGGAGAGRGAGCMTKAG	5' terminus
EukBshort_R	This study (modified from Medlin 1988)	R	CCTTCCGCAGGTTCACCTAC	3' terminus
LAOEukF	This study	F	CTGGTTGATCCTGCCAGTAKT	5' terminus
LAOEuk2F	This study	F	CTGGTTGATCCTGCCAGT	5' terminus
LAO18SF	This study	F	CGCGAANGGCTCATTANAWCAGC	5' terminus
LAOGiarF	This study	F	ACGGCTCAGGACAACGGTT	5' terminus
LAO1498R	This study	R	GGTTCACCTACGGANACCTTGTTA	3' terminus
LAOECR	This study	R	TCGTCTTCTCAGCGCCGGT	3' terminus
LAOEntCrypF	This study	F	GATTAAGCCATGCATGTSTAAG	5' terminus
LAO380F	This study	F	GGTTCGACTCCGGAGAG	5' terminus
LAOTW2F	This study	F	TGGATAACTGTAATRACTCT	5' terminus
LAOTW3R	This study	R	GACCTYACTAAACCATTCAATC	3' terminus

F, Forward primer; R, Reverse primer.

Supplementary Table 2. 18S V4 unresolved amplicon sequences.

Group	*ID 1*	*ID 2*	*ID 3*	ID 4	ID 5	ID 6
Blastocystis (n = 141)	*Blastocystis hominis*	*Blastocystis pythoni*				
	Blastocystis hominis	*Blastocystis ratti*				
Ciliophora (n = 172)	***Bandia smalesae***	***Bandia tammar***	***Triplumaria sukuna***			
	Triplumaria solea	*Triplumaria dvoinosi*				
Apicomplexa (n = 1,476)	*Besnoitia oryctofelisi*	*Besnoitia darlingi*				
	Cryptosporidium andersoni	*Cryptosporidium muris*				
	Cryptosporidium hominis	*Cryptosporidium parvum*				
	Cryptosporidium hominis	*Cryptosporidium parvum*				
	Cryptosporidium hominis	*Cryptosporidium parvum*				
	Eimeria tenella	*Eimeria necatrix*				
	Goussia janae	*Goussia pannonica*				
	Neospora caninum	***Hammondia heydorni***	***Hammondia triffittae***			
	Sarcocystis bovifelis	*Sarcocystis sinensis*				
	Sarcocystis levinei	*Sarcocystis cruzi*				
	Sarcocystis neurona	*Sarcocystis ramphastosi*				
	Sarcocystis tarandi	*Sarcocystis elongata*				
	Sarcocystis wobeseri	*Sarcocystis columbae*				
	Theileria buffeli	*Theileria orientalis*				
	Theileria buffeli	*Theileria orientalis*				
	Theileria orientalis	*Theileria annulata*				
	Theileria sergenti	*Theileria buffeli*				
	***Theileria* sp.**	***Babesia annae***				
Amoebozoa (n = 317)	*Acanthamoeba rhysodes*	*Acanthamoeba royreba*				
Acanthocephala (n = 72)	*Polymorphus minutus*	*Polymorphus obtusus*				

	Pomphorhynchus laevis	*Pomphorhynchus tereticollis*				
	Serrasentis nadakali	*Serrasentis sagittifer*				
Platyhelminthes (n = 531)	***Carmyerius spatiosus***	***Explanatum explanatum***	***Fischoederius elongatus***	***Gastrothylax crumenifer***	***Olveria bosi***	***Orthocoelium* sp.**
	Clonorchis sinensis	***Metorchis orientalis***	***Opisthorchis viverrini***			
	Cotylophoron cotylophorum	***Fischoederius cobboldi***	***Paramphistomum epiclitum***			
	Drepanocephalus auritus	***Drepanocephalus spathans***	***Mesorchis denticulatus***			
	Schistosoma curassoni	*Schistosoma mattheei*	*Schistosoma intercalatum*			
	Diphyllobothrium nihonkaiense	*Diphyllobothrium latum*				
	Echinostoma paraensei	*Echinostoma revolutum*				
	Nanophyetus salmincola	***Troglotrematidae* sp.**				
	Preptetos caballeroi	*Preptetos trulla*				
	Procerovum varium	*Procerovum cheni*				
	Raillietina beveridgei	*Raillietina australis*				
	Saccocoelium brayi	*Saccocoelium tensum*				
	Stichorchis subtriquetrus	***Paramphistomidae* sp.**				
	Trichobilharzia szidati	*Trichobilharzia ocellata*				
Nematoda (n = 460)	***Baylisascaris procyonis***	***Baylisascaris transfuga***	***Baylisascaris schroederi***	***Toxocara canis***	***Toxascaris leonina***	
	Viannaia didelphis	*Viannaia hamata*	*Viannaia minispicula*	*Viannaia viannai*		
	Cosmocercoides tonkinensis	*Cosmocercoides dukae*	*Cosmocercoides pulcher*			
	Haemonchus similis	*Haemonchus contortus*	*Haemonchus placei*			
	Philometra madai	*Philometra sawara*	*Philometra sciaenae*			
	Trichinella britovi	*Trichinella murrelli*	*Trichinella nativa*			
	Trichinella britovi	*Trichinella murrelli*	*Trichinella nativa*			
	Trichinella papuae	*Trichinella pseudospiralis*	*Trichinella zimbabwensis*			
	Iheringascaris inquies	***Raphidascaroides brasiliensis***				

	***Anisakis* sp.**	***Pseudoterranova decipiens***				
	Brugia malayi	*Brugia pahangi*				
	Camallanus lacustris	*Camallanus oxycephalus*				
	Dichelyne mexicanus	*Dichelyne robusta*				
	Dracunculus insignis	*Dracunculus lutrae*				
	Gongylonema aegypti	*Gongylonema neoplasticum*				
	Onchocerca volvulus	*Onchocerca cervicalis*				
	Philometra cyprinirutili	*Philometra ovata*				
	Procamallanus pacificus	***Spirocamallanus rarus***				
	Strongyloides callosciureus	*Strongyloides robustus*				
	Strongyloides papillosus	*Strongyloides venezuelensis*				
	Toxocara canis	*Toxocara cati*				
	Travassostrongylus callis	*Travassostrongylus orloffi*				
	Trichuris arvicolae	*Trichuris muris*				
	Trichuris discolor	*Trichuris ovis*				
	Troglostrongylus brevior	*Troglostrongylus wilsoni*				

Bolded rows, sequences assigned to more than one genus.

Supplementary Table 3. Parasite specimens and sources.

Organism	Sample type	Source	Catalog #
Echinorhynchus salmonis	Whole adult worms	UW Madison School of Veterinary Medicine, Dr. Tony Goldberg	NA
Hymenolepis diminuta	Whole adult worms	UW Madison School of Veterinary Medicine, Dr. Timothy Yoshino	NA
Taenia hydatigena	Cysts	Wisconsin Veterinary Diagnostic Lab	NA
Bertiella studeri	Proglottids	UW Madison School of Veterinary Medicine, Dr. Tony Goldberg	NA
Schistosoma mansoni Strain NMRI	DNA	BEI Resources	NR-28911
Ascaris suum	Whole adult worms	Wisconsin Veterinary Diagnostic Lab	NA
Dictyocaulus viviparous	Whole adult worms	Wisconsin Veterinary Diagnostic Lab	NA
Dirofilaria immitis Strain Missouri 2005	DNA	BEI Resources	NR-44348
Trichinella spiralis	DNA	USDA Animal Parasitic Diseases Laboratory	NA
Encephalitozoon cuniculi Strain CDC: V282	DNA	BEI Resources	NR-13510
Entamoeba histolytica Strain HK-9	DNA	BEI Resources	NR-175
Balamuthia mandrillaris CDC: V188	Axenic culture	BEI Resources	NR-46452
Acanthamoeba sp. Strain CDC: 12741:1	DNA	BEI Resources	NR-45611
Naegleria fowleri Strain CDC: V414	Axenic culture	BEI Resources	NR-46494
Leishmania major Strain NIH SD	DNA	BEI Resources	NR-48764
Trypanosoma cruzi Strain G	DNA	BEI Resources	NR-50238
Giardia lamblia Strain WB clone C6	DNA	BEI Resources	NR-15894
Plasmodium falciparum Strain D6	DNA	BEI Resources	MRA-398
Babesia sp. Strain MO1	DNA	BEI Resources	NR-50663
Toxoplasma gondii	DNA	UW Madison Department of Medical Microbiology and Immunology, Dr. Laura Knoll	NR-33509
Cryptosporidium hominis Strain TU502	DNA	BEI Resources	NR-2520
Blastocystis hominis Strain BT1	DNA	ATCC (American Type Culture Collection)	50608

NA, not applicable.

Supplementary Table 4. EukMix components and full-length 18S cloning primers.

	Organism	FWD primer*	REV primer*	Equimolar EukMix %	Log EukMix %
1	*Echinorhynchus salmonis*	EukA_F	EukB_R	6.25	0.125
2	*Hymenolepis diminuta*	LAOTW2F	LAOTW3R	6.25	12.5
3	*Ascaris suum*	LAO18SF	LAO1498R	6.25	2.5
4	*Dirofilaria immitis*	LAO18SF	LAO1498R	6.25	0.25
5	*Trichinella spiralis*	V3mod_F	EukBshort_R	6.25	25.0
6	*Encephalitozoon cuniculi*	V3mod_F	LAOECR	6.25	16.5
7	*Entamoeba histolytica*	LAOEuk2F	EukB_R	6.25	12.5
8	*Balamuthia mandrillaris*	EukA_F	EukB_R	6.25	2.5
9	*Naegleria fowleri*	LAO380F	LAO1498R	6.25	0.0625
10	*Giardia intestinalis*	LAO380F	EukB_R	6.25	0.125
11	*Leishmania major*	LAOEukF	EukB_R	6.25	0.125
12	*Plasmodium falciparum*	EukA_F	EukB_R	6.25	2.5
13	*Babesia* sp. strain MO1	EukA_F	EukB_R	6.25	0.0625
14	*Toxoplasma gondii*	EukA_F	EukB_R	6.25	25
15	*Cryptosporidium hominis*	EukA_F	LAO1498R	6.25	0.25
16	*Blastocystis hominis*	LAOEukF	LAO1498R	6.25	0.25

*See Supplementary Table 1 for primer sequences and references.

Supplementary Table 5. EukMix community standard metabarcoding mean fold distance from theoretical: exact *P*-values.

Primer 1	vs	Primer 2	Community Standard	*P*-value*
Owens 29F		Owens 2-2bF	Equimolar EukMix	0.029
Owens 29F		Stoack TAR	Equimolar EukMix	0.0017
Owens 29F		Hadz. 566	Equimolar EukMix	0.0006
Owens 29F		Bates 515	Equimolar EukMix	0.0003
Owens 29F		Owens 2-2bF	Log EukMix	0.7057
Owens 29F		Stoack TAR	Log EukMix	0.0063
Owens 29F		Hadz. 566	Log EukMix	0.0003
Owens 29F		Bates 515	Log EukMix	0.0034

*Wilcoxon matched-pairs signed rank test, 2-tailed.

Supplementary Table 6. VESPA vs microscopy MiSeq run metrics.

Library ID	SRA accession	Sample type	Raw reads	Reads post-quality filter	% lost in filter
Human01	SAMN33744948	Human fecal	62,512	53239	9.45%
Human02	SAMN33744949	Human fecal	32,755	30,195	7.82%
Human03	SAMN33744950	Human fecal	223,911	206,999	7.55%
Human04	SAMN33744951	Human fecal	43,371	39,228	9.55%
Human05	SAMN33744952	Human fecal	116,016	106,130	8.52%
Human06	SAMN33744953	Human fecal	24,095	22,204	7.85%
Human07	SAMN33744954	Human fecal	55,882	50,772	9.14%
Human08	SAMN33744955	Human fecal	80,184	72,324	9.80%
Human09	SAMN33744956	Human fecal	35,824	32,808	8.42%
Human10	SAMN33744957	Human fecal	30,176	27,645	8.39%
Human11	SAMN33744958	Human fecal	78,021	72,165	7.51%
Human12	SAMN33744959	Human fecal	123,564	112,774	8.73%
NHP1	SAMN33744960	Nonhuman primate fecal	37,377	35,637	4.65%
NHP2	SAMN33744961	Nonhuman primate fecal	98,953	92,910	6.11%
NHP3	SAMN33744962	Nonhuman primate fecal	287,932	269,181	6.51%
NHP4	SAMN33744963	Nonhuman primate fecal	56,002	52,080	7.00%
NHP5	SAMN33744964	Nonhuman primate fecal	28,351	26,874	5.21%
NHP6	SAMN33744965	Nonhuman primate fecal	104,900	97,907	6.67%
NHP7	SAMN33744966	Nonhuman primate fecal	28,409	26,415	7.02%
NHP8	SAMN33744967	Nonhuman primate fecal	25,764	23,788	7.67%
NHP9	SAMN33744968	Nonhuman primate fecal	29,434	27,018	8.21%
NHP10	SAMN33744969	Nonhuman primate fecal	58,005	53,206	8.27%
NHP11	SAMN33744970	Nonhuman primate fecal	44,422	39,862	10.26%
NHP12	SAMN33744971	Nonhuman primate fecal	36,887	33,991	7.85%
NHP13	SAMN33744972	Nonhuman primate fecal	55,101	49,958	9.33%
NHP14	SAMN33744973	Nonhuman primate fecal	34,701	31,934	7.97%
NHP15	SAMN33744974	Nonhuman primate fecal	64,954	60,237	7.26%
NHP16	SAMN33744975	Nonhuman primate fecal	50,839	47,371	6.82%
NHP17	SAMN33744976	Nonhuman primate fecal	75,005	68,826	8.24%
NHP18	SAMN33744977	Nonhuman primate fecal	76,770	70,964	7.56%
NHP19	SAMN33744978	Nonhuman primate fecal	46,543	44,239	4.95%

NHP20	SAMN33744979	Nonhuman primate fecal	40,031	37,507	6.31%
NHP21	SAMN33744980	Nonhuman primate fecal	39,344	36,571	7.05%
NHP22	SAMN33744981	Nonhuman primate fecal	29,797	27,118	8.99%
NHP23	SAMN33744982	Nonhuman primate fecal	36,615	33,891	7.44%
NHP24	SAMN33744983	Nonhuman primate fecal	84,056	76,577	8.90%
NHP25	SAMN33744984	Nonhuman primate fecal	27,672	26,198	5.33%
NHP26	SAMN33744985	Nonhuman primate fecal	32,150	28,996	9.81%
NHP27	SAMN33744986	Nonhuman primate fecal	157,483	144,045	8.53%
NHP28	SAMN33744987	Nonhuman primate fecal	31,830	29,320	7.88%
NHP29	SAMN33744988	Nonhuman primate fecal	41,127	37,816	8.05%
NHP30	SAMN33744989	Nonhuman primate fecal	60,491	55,710	7.90%
NHP31	SAMN33744990	Nonhuman primate fecal	74,435	67,968	8.69%
NHP32	SAMN33744991	Nonhuman primate fecal	59,136	54,146	8.44%
NHP33	SAMN33744992	Nonhuman primate fecal	35,473	32,346	8.82%
NHP34	SAMN33744993	Nonhuman primate fecal	39,545	36,508	7.68%
NHP35	SAMN33744994	Nonhuman primate fecal	33,505	31,048	7.33%
NHP36	SAMN33744995	Nonhuman primate fecal	44,082	41,003	6.98%
NHP37	SAMN33744996	Nonhuman primate fecal	59,451	54,867	7.71%
NHP38	SAMN33744997	Nonhuman primate fecal	14,879	13,872	6.77%
NHP39	SAMN33744998	Nonhuman primate fecal	71,275	64,595	9.37%
NHP40	SAMN33744999	Nonhuman primate fecal	62,042	57,199	7.81%

VESPA Protocol

March 2023

Contents

1- Starting material
2- gDNA extraction
3- 18S V4 Amplicon PCR
4- Amplicon cleanup
5- Indexing PCR
6- Library cleanup
7- Quantification and size determination
8- Pooling and sequencing

1- Starting material

Starting material can be fresh, freshly frozen (no buffer), or stored ~1:1 in RNA later.

Sample types tested:

Feces	Vomit	Stomach- contents
Intestine- tissue	Intestine- contents	Environmental
Entamoeba cysts	Whole helminths	Tapeworm proglottids/cysts

2- gDNA extraction

Use

- Qiagen DNeasy PowerLyzer PowerSoil Kit (catalog #12855-5)

according to manufacturer's instructions.

Weigh out up to .20 g of input feces or .25 g of input for all other sample types.

Elute in 100 µl C6 buffer (included in kit) and store at -20 °C.

3- 18S V4 Amplicon PCR

Set up amplicon PCR reactions *in triplicate.*

Use

- Invitrogen Platinum II Hot Start 2X PCR Master Mix (Catalog # 14000012)

with the following reaction and cycling conditions:

Reaction component	Final Conc.	1 x 12.5 µl rxn. (µl)
2X Platinum II HotStart PCR Master Mix*	1X	6.0
10 µM Forward primer	0.2 µM	0.25
10 µM Reverse primer	0.2 µM	0.25
Platinum II GC Enhancer*	NA	2.5
Nuclease-free water*	NA	2.5
~10 ng/µl gDNA	0.8 ng/µl	1.0

12.5 µl

*Included in Master Mix Kit

Primers	Nextera adapter sequence **Locus-specific sequence**
Forward: 29_F	TCGTCGGCAGCGTCAGATGTGTATAAGAGACAG**AGCAGCCGCGGTAATTCC**
Reverse: 21b8_I_R	GTCTCGTGGGCTCGGAGATGTGTATAAGAGACAG**TCCGTCAATTYCTTIAASTTTC**

Step	Temp °C	Time	Cycles
Activation	94	2 min	1
Denaturation	94	15 sec	30
Annealing	60	15 sec	
Extension	68	15 sec	
Final hold	4	hold	

4- Amplicon cleanup

Use

- Beckman Coulter Ampure XP beads (catalog #A63880)

and

- Magnetic particle separator (MPC).

Always make 75% Ethanol immediately prior to use.

1- Shake Ampure XP beads at room temperature for > 30 minutes prior to use.
2- *Pool* all 3 PCR reactions into a single plate or tube and mix by pipetting (~37.5 µl).
3- Remove 7.5 µl and store at -20 °C if you would like to visualize bands on a gel (~30 µl).
4- Add AMPure XP beads for **0.8X RATIO** (*e.g.* 24 µl beads per 30 µl product).
5- Gently pipette up and down 15 times.
6- Incubate at room temperature for 5 minutes.
7- Put tubes on MPC and incubate at room temperature for 2 minutes.
8- Remove and discard supernatant.
9- With tubes on MPC, add 175 µl of 75% ethanol.
10- Wait >1 minute.
11- Remove and discard supernatant.
12- Add 175 µl of 75% ethanol.
13- Wait >1 minute.
14- Remove and discard supernatant.
15- Remove all ethanol with P20 tips.
16- With tubes on MPC, let the pellet air-dry for 5 minutes.
17- Add **47 µl of Tris pH 8.5.**

18- Remove tubes from MPC and gently pipette up and down to resuspend beads.
19- Incubate at room temperature for 2 minutes.
20- Put tubes on MPV and incubate at room temperature for 2 minutes.
21- Carefully transfer **45 µl** of supernatant to a new PCR tubes or plate.

5- Indexing PCR

Set up Indexing PCR reactions *on ice.*

Use

- Roche KAPA HiFi HotStart ReadyMix (catalog #KK2601)

and

- IDT for Illumina Nextera DNA Unique Dual Indexes (catalog #20027215)

with the following reaction and cycling conditions:

Reaction component	1 x 12.5 µl rxn. (µl)
2X KAPA HiFi HotStart ReadyMix	6.0
Nextera Unique Dual Index	2.5
Nuclease-free water	3.0
Clean amplicons in Tris pH 8.5	1.0
	12.5 µl

<table>
<tr><th>Step</th><th>Temp °C</th><th>Time</th><th>Cycles</th></tr>
<tr><td>Activation</td><td>95 °C</td><td>3 min</td><td>1</td></tr>
<tr><td>Denaturation</td><td>95 °C</td><td>30 sec</td><td rowspan="3">10</td></tr>
<tr><td>Annealing</td><td>55 °C</td><td>30 sec</td></tr>
<tr><td>Extension</td><td>72 °C</td><td>30 sec</td></tr>
<tr><td>Final extension</td><td>72 °C</td><td>5 min</td><td>1</td></tr>
<tr><td>Final hold</td><td>4 °C</td><td>hold</td><td></td></tr>
</table>

6- Library cleanup

Use

- Beckman Coulter Ampure XP beads (catalog #A63880)

and

- Magnetic particle separator (MPC).

Always make 75% Ethanol immediately prior to use.

1- Shake Ampure XP beads at room temperature for > 30 minutes prior to use.
2- Add AMPure XP beads for **0.8X RATIO** (*e.g.* 9.6 µl beads per 12.5 µl PCR product).
3- Gently pipette up and down 15 times.

4- Incubate at room temperature for 5 minutes.
5- Put tubes on MPC and incubate at room temperature for 2 minutes.
6- Remove and discard supernatant.
7- With tubes on MPC, add 175 µl of 75% ethanol.
8- Wait >1 minute.
9- Remove and discard supernatant.
10- Add 175 µl of 75% ethanol.
11- Wait >1 minute.
12- Remove and discard supernatant.
13- Remove all ethanol with P20 tips.
14- With tubes on MPC, let the pellet air-dry for 5 minutes.
15- Add **22 µl of Tris pH 8.5.**
16- Remove tubes from MPC and gently pipette up and down to resuspend beads.
17- Incubate at room temperature for 2 minutes.
18- Put tubes on MPV and incubate at room temperature for 2 minutes.
19- Carefully transfer **20 µl** of supernatant to a new PCR tubes or plate.

7- Quantification and size determination

Use

- Invitrogen Qubit Fluorimeter and dsDNA High-Sensitivity Assay Kit (catalog #Q33230)

and

- Agilent Bioanalyzer and Agilent High Sensitivity DNA Kit (catalog #5067-4626)

according to manufacturer's instructions.

Measure the concentration of each library using a Qubit fluorometer and 3 µl of each library.

Measure the size of each library or a representative subset of libraries using an Agilent Bioanalyzer and 1 µl of a 1 ng/µl dilution (in Tris pH 8.5) of the library for a total of 1 ng.

8- Pooling and sequencing

Requirements for core facility submission/in-house sequencing will determine pooling specifics.
Run on an Illumina MiSeq instrument, 300 x 300 cycle chemistry, and add 10 – 20% PhiX.
