# Supplementary material for: VESPA: an optimized protocol for accurate metabarcoding-based characterization of vertebrate eukaryotic endosymbiont and parasite assemblages
Source: Nat Commun. 2024 Jan 9;15:402. doi: 10.1038/s41467-023-44521-3 (PMC10776621; doi:10.1038/s41467-023-44521-3)
Supplement: Supplementary file 4 — Reporting Summary [file 41467_2023_44521_MOESM4_ESM.pdf]

Corresponding author(s): Tony L. Goldberg
Leah A. Owens

Last updated by author(s): Dec 5, 2023

Reporting Summary

Nature Portfolio wishes to improve the reproducibility of the work that we publish. This form provides structure for consistency and transparency in reporting. For further information on Nature Portfolio policies, see our Editorial Policies and the Editorial Policy Checklist.

Statistics

For all statistical analyses, confirm that the following items are present in the figure legend, table legend, main text, or Methods section.

n/a	Confirmed	
☐	☒	The exact sample size (*n*) for each experimental group/condition, given as a discrete number and unit of measurement
☐	☒	A statement on whether measurements were taken from distinct samples or whether the same sample was measured repeatedly
☐	☒	The statistical test(s) used AND whether they are one- or two-sided<br>*Only common tests should be described solely by name; describe more complex techniques in the Methods section.*
☒	☐	A description of all covariates tested
☒	☐	A description of any assumptions or corrections, such as tests of normality and adjustment for multiple comparisons
☐	☒	A full description of the statistical parameters including central tendency (e.g. means) or other basic estimates (e.g. regression coefficient) AND variation (e.g. standard deviation) or associated estimates of uncertainty (e.g. confidence intervals)
☐	☒	For null hypothesis testing, the test statistic (e.g. F, t, r) with confidence intervals, effect sizes, degrees of freedom and P value noted<br>*Give P values as exact values whenever suitable.*
☒	☐	For Bayesian analysis, information on the choice of priors and Markov chain Monte Carlo settings
☒	☐	For hierarchical and complex designs, identification of the appropriate level for tests and full reporting of outcomes
☒	☐	Estimates of effect sizes (e.g. Cohen's d, Pearson's r), indicating how they were calculated

Our web collection on statistics for biologists contains articles on many of the points above.

Software and code

Policy information about availability of computer code

Data collection	MEGA v.11, arb v.7.0, OBITools in Python v.2.7, SILVA TestPrime v.1.0, SILVA TestProbe v.3.0
Data analysis	CLC v.10.2, R v.3.6.3, Graphpad Prism v.8.4.3, DADA2 v.1.16.0, TaxMan server

For manuscripts utilizing custom algorithms or software that are central to the research but not yet described in published literature, software must be made available to editors and reviewers. We strongly encourage code deposition in a community repository (e.g. GitHub). See the Nature Portfolio guidelines for submitting code & software for further information.

Data

Policy information about availability of data

All manuscripts must include a data availability statement. This statement should provide the following information, where applicable:

- Accession codes, unique identifiers, or web links for publicly available datasets
- A description of any restrictions on data availability
- For clinical datasets or third party data, please ensure that the statement adheres to our policy

The DNA sequencing data generated in this study have been deposited in the National Center for Biotechnology Information (NCBI) Sequence Read Archive under BioProject ID PRJNA944233 https://www.ncbi.nlm.nih.gov/sra/PRJNA944233. The in silico PCR (% primer coverage) data, mock community standard analysis data, off-target read abundance data, and percent read abundance data generated in this study are provided in the Source Data file. Publicly available databases used in this study are: PR2 reference sequence database v 5.0.1 DOI 10.5281/zenodo.7805244, the SILVA v128 and v132 dada2 formatted 18s 'train sets' DOI 10.5281/

zenodo.1447329, and the SILVA SSU rRNA v138.1 Ref NR database https://www.arb-silva.de/fileadmin/arb_web_db/release_138_1/ARB_files/SILVA_138.1_SSURef_NR99_12_06_20_opt.arb.gz licensed under Creative Commons Attribution 4.0 https://creativecommons.org/licenses/by/4.0/.

Research involving human participants, their data, or biological material

Policy information about studies with human participants or human data. See also policy information about sex, gender (identity/presentation), and sexual orientation and race, ethnicity and racism.

Reporting on sex and gender	Human fecal samples were excess material from a concluded study that were completely de-identified prior to use in this study.
Reporting on race, ethnicity, or other socially relevant groupings	Human fecal samples were excess material from a concluded study that were completely de-identified prior to use in this study.
Population characteristics	Human fecal samples were excess material from a concluded study that were completely de-identified prior to use in this study.
Recruitment	Human fecal samples were excess material from a concluded study that were completely de-identified prior to use in this study.
Ethics oversight	IRB approval for original studies using human fecal samples: IVIC IRB #DIR-0609/1542/2015, UW Madison IRB #2013-1463.

Note that full information on the approval of the study protocol must also be provided in the manuscript.

Field-specific reporting

Please select the one below that is the best fit for your research. If you are not sure, read the appropriate sections before making your selection.

☒ Life sciences ☐ Behavioural & social sciences ☐ Ecological, evolutionary & environmental sciences

For a reference copy of the document with all sections, see nature.com/documents/nr-reporting-summary-flat.pdf

Life sciences study design

All studies must disclose on these points even when the disclosure is negative.

Sample size	Sample sizes for fecal experiments were chosen opportunistically based on number of samples available from previous studies and no statistical method was used to predetermine sample size.
Data exclusions	No samples and no data were excluded from analysis of any experiment.
Replication	For PCR and metabarcoding experiments using purified DNA from single organisms or mock community standards, each experiment was successfully repeated with the same outcomes. Fecal amplicon PCR was performed successfully in triplicate and amplicons were pooled prior to library preparation.
Randomization	Randomization was not used in this study because all samples were used for the same analysis, which was a comparison between methods.
Blinding	For PCR and metabarcoding experiments using purified DNA from single organisms or mock community standards, investigators were blinded to primer and template DNA identity and gels were scored by two independent investigators. For metabarcoding of clinical fecal samples, investigators were blinded to host species and infection status.

Reporting for specific materials, systems and methods

We require information from authors about some types of materials, experimental systems and methods used in many studies. Here, indicate whether each material, system or method listed is relevant to your study. If you are not sure if a list item applies to your research, read the appropriate section before selecting a response.

Materials & experimental systems

n/a	Involved in the study
☒	☐ Antibodies
☒	☐ Eukaryotic cell lines
☒	☐ Palaeontology and archaeology
☐	☒ Animals and other organisms
☒	☐ Clinical data
☒	☐ Dual use research of concern
☒	☐ Plants

Methods

n/a	Involved in the study
☒	☐ ChIP-seq
☒	☐ Flow cytometry
☒	☐ MRI-based neuroimaging

Animals and other research organisms

Policy information about studies involving animals; ARRIVE guidelines recommended for reporting animal research, and Sex and Gender in Research

Laboratory animals	This study did not involve laboratory animals.
Wild animals	Fecal samples from semi-wild red capped mangabeys (Cercocebus torquatus) living in a sanctuary that were used in this study were excess material from a concluded study and completely de-identified prior to use.
Reporting on sex	Sex was not considered in this study. Animal samples were excess material from a concluded study with no accompanying demographic information and were completely de-identified prior to use.
Field-collected samples	Field-collected fecal samples from semi-wild red capped mangabeys (Cercocebus torquatus) used in this study were excess material from a concluded study and completely de-identified prior to use. No demographic or identifying information was provided. For single organisms used for DNA extraction (parasitic worms and protozoa), samples (whole adult worms, cysts, proglottids, axenic cultures, or purified DNA) were obtained from expert parasitologists or from reputable reagent repositories (for sample details including sources see Supplementary Table 3).
Ethics oversight	IACUC approval for original study using red capped mangabey fecal samples: UW-Madison protocol # V1490. There was no ethics oversight required for the use of invertebrate and protozoan organisms in this study.

Note that full information on the approval of the study protocol must also be provided in the manuscript.
